# Circ_0036412 affects the proliferation and cell cycle of hepatocellular carcinoma via hedgehog signaling pathway

**DOI:** 10.1186/s12967-022-03305-x

**Published:** 2022-04-05

**Authors:** Liyan Wang, Bin Li, Xiaoyuan Yi, Xuhua Xiao, Qinghua Zheng, Lei Ma

**Affiliations:** Department of Gastroenterology, Affiliated Hospital of Guilin Medical College, No. 15 Lequn Road, Xiufeng District, Guilin, 541001 Guangxi China

**Keywords:** HCC, circ_0036412, GLI2, Hedgehog signaling pathway

## Abstract

**Background:**

Hepatocellular carcinoma (HCC), as the most common type of liver cancer, is characterized by high recurrence and metastasis. Circular RNA (circRNA) circ_0036412 was selected for studying the underlying mechanisms of HCC.

**Methods:**

Quantitative real time-polymerase chain reaction (qRT-PCR) and western blot analyzed gene and protein expression. Functional experiments evaluated HCC cell proliferation, apoptosis and cell cycle in vitro. In vivo experiments detected HCC carcinogenesis in vivo. Fluorescence in situ hybridization (FISH) assays evaluated the subcellular distribution. Luciferase reporter, Chromatin immunoprecipitation (ChIP), DNA pulldown, RNA-binding protein immunoprecipitation (RIP), and RNA pulldown assays detected the underlying mechanisms.

**Results:**

Circ_0036412 is overexpressed in HCC cells and features circular structure. PRDM1 activates circ_0036412 transcription to regulate the proliferation and cell cycle of HCC cells in vitro. Circ_0036412 modulates Hedgehog pathway. GLI2 propels HCC growth in vivo. Circ_0036412 up-regulates GLI2 expression by competitively binding to miR-579-3p, thus promoting the proliferation and inhibiting cell cycle arrest of HCC cells. Circ_0036412 stabilizes GLI2 expression by recruiting ELAVL1. Circ_0036412 propels the proliferation and inhibits cell cycle arrest of HCC cells in vitro through Hedgehog pathway.

**Conclusions:**

Circ_0036412 affects the proliferation and cell cycle of HCC via Hedgehog signaling pathway. It offers an insight into the targeted therapies of HCC.

**Graphical Abstract:**

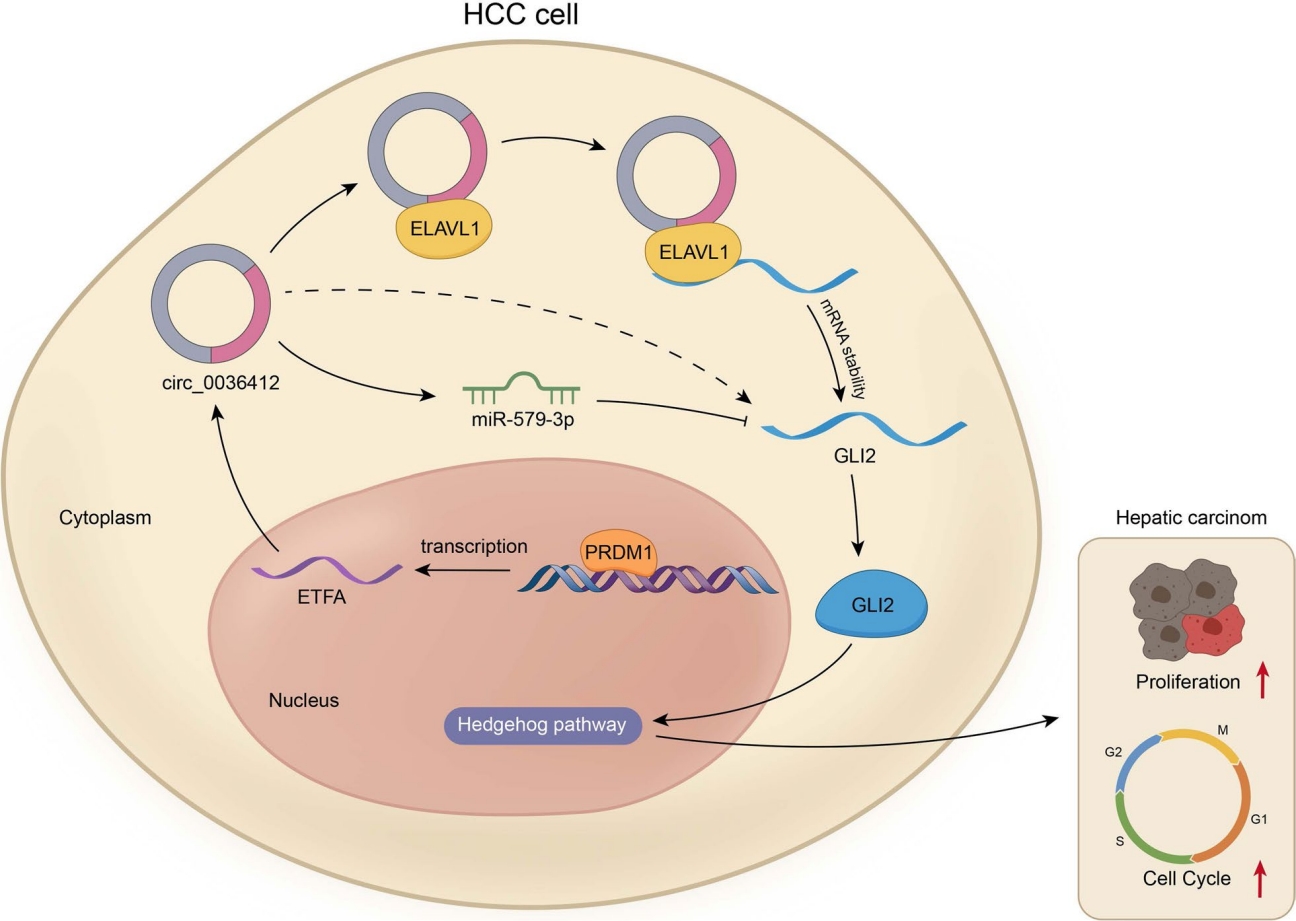

**Supplementary Information:**

The online version contains supplementary material available at 10.1186/s12967-022-03305-x.

## Background

Hepatocellular carcinoma (HCC) is the most common primary liver carcinoma, the mortality of which ranks second among all the cancers [1]. The main risk factor for HCC is cirrhosis resulting from chronic hepatitis B or hepatitis C, which serves as the causes of the majority of HCC cases [2]. Also, immune-related hepatitis, alcohol addiction, diabetes, nonalcoholic fatty liver disease and dietary toxins are likely to increase HCC incidence [3]. Various therapeutic methods have been developed for HCC, including surgery, interventional embolization, radiofrequency ablation (RFA) as well as chemotherapy [4, 5]. Despite the development of methods, the high rates of recurrence and metastasis limit the treatment of HCC patients; therefore, the accurate prediction of the recurrence and metastasis of HCC is crucial to improving the prognosis and overall survival of HCC patients [6, 7]. The current methods are still hard to predict HCC recurrence and metastasis because of its complexity and heterogeneity. Thus, it’s urgent to investigate reliable biomarkers for prediction.

Circular RNA (circRNA), as a class of non-coding RNA, features a covalently closed loop. Many circRNAs have been reported to be aberrantly expressed in various cancers [8]. These aberrantly expressed circRNAs serve as important regulators in multiple cancers, including HCC. For instance, circRNA-5692 suppresses the malignant progression of HCC via sequestering miR-328-5p to up-regulate DAB2IP expression [9]; circRNA-SORE improves the stability of YBX1 by mediating sorafenib resistance in HCC [10]; circRNA_104348 facilitates HCC malignancy by regulating miR-187-3p/RTKN2 axis [2]; circRHOT1 propels the development of HCC via initiating the expression of NR2F6 [11]; and circRNA Cdr1as as a competitive endogenous RNA (ceRNA) sponges miR-1270 to up-regulate AFP expression, thereby facilitating the development of HCC [12].

CircETFA (also termed as circ_0036412) promotes HCC progression via sponging miR-612 and recruiting EIF4A3 to increase CCL5 expression [13]. The host gene of circ_0036412, ETFA has been reported to be associated with Multiple Acyl-CoA Dehydrogenase Deficiency (MADD) [14]. Moreover, NBPF4 regulates ETFA, which is associated with EZH2, to attenuate the progression of colorectal cancer [15].

Hedgehog signaling pathway, with the Patched (PTCH) and Smoothened (SMO) multitransmembrane proteins, has been found to play an important role in vertebrate embryonic development and tumorigenesis [16]. It has been found that Hedgehog pathway is involved in HCC. CircZNF609 facilitates the proliferation, metastasis and stemness of HCC via the activation of the Hedgehog pathway by regulating the expressions of miR-15a-5p, miR-15b-5p and GLI2 [17]; KLF2 attenuates the progression of HCC by negatively modulating the Hedgehog pathway [18]; Sesn3 ablation propels carcinogen-induced HCC via modulation of the Hedgehog pathway [19]; MIRLET7BHG facilitates the progression of HCC through the activation of hepatic stellate cells by activating Hedgehog pathway [20]; and circIPO11 drives the proliferation of HCC via Hedgehog signaling pathway [21]. However, the correlation of Hedgehog signaling pathway with circ_0036412 hasn’t been explored before.

Our study aimed to research the regulatory role of circ_0036412 and Hedgehog signaling pathway underlying the progression of HCC. In hope to find out the reliable biomarkers for HCC, we adopted bioinformatics for the screening of the differentially expressed circRNAs in HCC specimens. We identified circ_0036412 as the focus of our study.

## Methods

### Cell culture and treatment

Human normal liver cell line (THLE-2) and HCC cell lines (HuH-7, Hep 3B and Li-7) were all procured from ATCC, and human embryonic kidney cell line (293 T) was obtained from National Institutes for Food and Drug Control. HuH-7 and Hep 3B cells were maintained in MEM-EBSS added with 10% fetal bovine serum (FBS). Li-7 cells were cultured in RPMI 1640 (w/o Hepes) + 10% FBS. THLE-2 cells were maintained in RPMI 1640 with the supplementation of 10% FBS and 1% penicillin and streptomycin. 293 T cells were cultured in RPMI 1640 supplemented with 10% FBS. All the cell lines were maintained with 5% CO_2_ at 37 °C.

### Plasmids and transfection

Full-length sequences of PRDM1, circ_0036412 and GLI2 were inserted into pcDNA3.1 vectors for overexpression, with pcDNA3.1 vector being used as the negative control (NC). Small interfering RNAs (siRNAs) targeting PRDM1, circ_0036412, GLI2 and ELAVL1 were constructed to create interference vectors, with si-NC as NC. Lipofectamine 2000 was used for transfection.

### Quantitative real time-polymerase chain reaction (RT-qPCR)

The total RNA was subjected to isolation from cells using Trizol. Next, the samples were reverse-transcribed into complementary DNA (cDNA), followed by the qPCR analysis. According to the method of 2^−ΔΔCt^, the results were calculated. Bio-repeats were performed in triplicate.

### Ribonuclease R (RNase R) and actinomycin D (ActD) treatments

RNase R and ActD treatments were implemented to evaluate the stability of circ_0036412 and ETFA. The cells were seeded into 6-well plates, followed by the treatment with RNase R or 2 μg/mL ActD. The levels of circ_0036412 and ETFA were measured by RT-qPCR in RNase R-treated cells. The levels of circ_0036412 and ETFA were measured by RT-qPCR every 6 h till 24 h in ActD-treated cells.

### Agarose gel electrophoresis

Agarose powder and TAE solution were added in a conical bottle, followed by being heated for 3–5 min. Afterwards, the boiled agarose gel was added with 1 µM nucleic acid. The gel was poured into the mold and then solidified. After the solidification, the gel was treated with 0.5 × TAE solution in the electrophoresis tank. Subsequently, the PCR product was mixed with DNA ladder and then added to gel, followed by constant-pressure 90 V electrophoresis for 20–30 min. Later, the gel was observed by an ultraviolet lamp. RNase R was used to treat cDNA. Bio-repeats were performed in triplicate.

### Western blot

The total protein was extracted from cells. The cell samples were subjected to separation via SDS-PAGE. After being transferred onto polyvinylidene fluoride (PVDF) membrane, the samples were blocked by 5% defatted milk for 1 h. The blocked samples were subjected to incubation with the primary antibodies overnight at 4 °C, followed by being incubated with secondary antibodies. The results were then visualized and recorded. The primary antibodies include Anti-PRDM1, Anti-GLI2, Anti-IGF2BP2, Anti-ELAVL1, Anti-Histone H3, Anti-CCND1 and Anti-β-actin. Histone H3 and β-actin were used as the internal references. Bio-repeats were performed in triplicate.

### Flow cytometry analysis

The cells were washed in PBS, followed by the centrifugation. After the addition of precooled ethanol, the samples were subjected to centrifugation again, followed by the washes. Subsequently, the samples were stained with 0.5 mL PI/RNase. The FACSCalibur system was adopted for the analysis of the samples. Data analysis was conducted using FlowJo software.

### 5-Ethynyl-2′-deoxyuridine (EdU) assay

The HCC cells were seeded into 12-well plates. After the transfection, the cells treated with 50 μmol/L EdU reagent for 2 h. After being washed in PBS, the samples were treated with 4% phosphate-buffered paraformaldehyde for fixation. After the staining, a fluorescence microscope was employed to count EdU-positive cells. Bio-repeats were conducted in triplicate.

### Terminal deoxynucleotidyl transferase dUTP nick end labeling (TUNEL) assay

After the fixation with paraformaldehyde, transfected HCC cells were subjected to treatment with TUNEL kit under the manufacturer’s instructions. Fluorescence microscope was used to detect cell number of TUNEL-positive. Bio-repeats were performed in triplicate.

### Cell counting kit-8 (CCK-8) assay

CCK-8 assay was conducted using CCK-8 kit as instructed by the manufacturer’s protocol. Transfected HCC cells were placed into 96-well culture plates. Next, all the transfected cells underwent 0, 24, 48 and 72 h of incubation, along with the supplementation of 100 μL CCK-8 solution to each well. CCK-8 solution was incubated with the cells for another 4 h. A spectrophotometer was adopted to evaluate OD value at the wavelength of 450 nm. Bio-repeats were implemented in triplicate.

### Fluorescence in situ hybridization (FISH) assay

The cells were seeded in 12-well plates prior to FISH assay. After being fixed in 4% paraformaldehyde and permeabilized in 0.5% Triton X-100, the cells were subjected to the incubation with digoxigenin-labeled (DIG-labeled) FISH probe targeting circ_0036412 and GLI2. The antibody of DIG was used to detect signal. A laser confocal microscope was applied to acquire images. DAPI solution was adopted for counterstaining cell nucleus. Bio-repeats were implemented in triplicate.

### In vivo experiments

The BALB/c nude mice (male, 6 weeks old) were commercially obtained from Model Animal Research Center of Nanjing University. Firstly, the knockdown vector targeting GLI2 was transfected into HCC cells. After 48 h, transfected HCC cells were subjected to subcutaneously injection into the nude mice. The tumor volume was measured every 3 days. Twenty-eight days after the injection, the tumors were resected from the mice and then weighed.

In vivo lung metastasis experiments were performed as described before [22]. HCC cells were injected into nude BALB/c mice via hydrodynamic tail vein. Forty days later, the lung tissues excised from the mice were subjected to H&E staining for visualization of the metastatic lesions within the lung. The liver tumor foci were measured via microscopy. The animal experiments were approved by Affiliated Hospital of Guilin Medical College.

### Luciferase reporter assay

The full-length sequence of circ_0036412 or GLI2 3′ UTR sequence with miR-579-3p binding site was subcloned into pmirGLO vector to construct pmirGLO-circ_0036412/GLI2 3’ UTR WT. Circ_0036412 or GLI2 3’ UTR sequence containing mutant binding sites of miR-579-3p was used to construct pmirGLO-circ_0036412/GLI2 3’ UTR MUT. Empty pmirGLO vector was used as NC. The reporter vectors were co-transfected with miR-579-3p mimics or mimics NC into 293 T cells. The sequence of ETFA promoter was subcloned into pGL3 vector to construct pGL3-ETFA promoter WT. We mutated 1505–1515 site in the sequence of IL-10 promoter, converting CTCTCTCTCTC into CTCTCCCTCTC. Subsequently, ETFA promoter sequence containing this mutated binding site was subcloned into pGL3 vector to construct pGL3-ETFA promoter MUT. The above reporter vectors were co-transfected with pcDNA3.1-PRDM1 or pcDNA3.1 into 293 T cells. The empty pGL3 vector was used as NC. The relative luciferase activity was determined using Dual-Luciferase Reporter Assay System. Bio-repeats were performed in triplicate.

### Chromatin immunoprecipitation (ChIP) assay

HCC cells were crosslinked for 5 min at room temperature, followed by being washed in cold PBS. Next, the samples were subjected to centrifugation at 2000 rpm for 5 min. After being lysed in RIPA Lysis Buffer, the lysate was incubated with Anti-IgG and Anti-PRDM1 respectively. After the incubation, the precipitate was treated with 5 mL NaCl for de-crosslinking. The samples were then incubated with RNase A, prior to the analysis of qRT-PCR.

### RNA-binding protein immunoprecipitation (RIP) assays

Imprint® RNA Immunoprecipitation Kit was used for RIP assay. The cell samples were lysed in RIPA Lysis Buffer. The magnetic beads were conjugated with Anti-AGO2, Anti-ELAVL1 and Anti-IgG, followed by the incubation with the lysate. Enrichment of certain RNAs was determined by RT-qPCR, with IgG as NC. Bio-repeats were performed in triplicate.

### RNA/DNA pulldown assay

Structure buffer was added to biotinylated circ_0036412/miR-579-3p WT/miR-579-3p MUT/GLI2 3′UTR Anti-sense/GLI2 3′UTR sense WT/GLI2 3’UTR sense MUT/ETFA Promoter/ETFA Promoter MUT for the formation of secondary structure. Afterwards, the samples were heated and ice-bathed for denaturation, followed by the incubation with streptavidin beads at 4 °C for 2 h. The cell lysate was prepared to set up Input, Bio-NC and Bio-RNAs. Subsequently, these groups were incubated with the magnetic beads at 4 °C for one night. Western blot or qRT-PCR was applied to analyze the pulldown products. Bio-repeats were conducted in triplicate.

### Statistical analysis

The data in the experiments were presented as mean ± standard deviation (SD), with SPSS software as the tool for data analysis. Student’s t test was applied to compare differences between two groups, while one-way/two-way analysis of variance (ANOVA) to compare differences between more than two groups. P value lower than 0.05 was considered as the criterion for statistical significance.

## Results

### The characterization of circ_0036412 in HCC cells

**Fig. 1:**
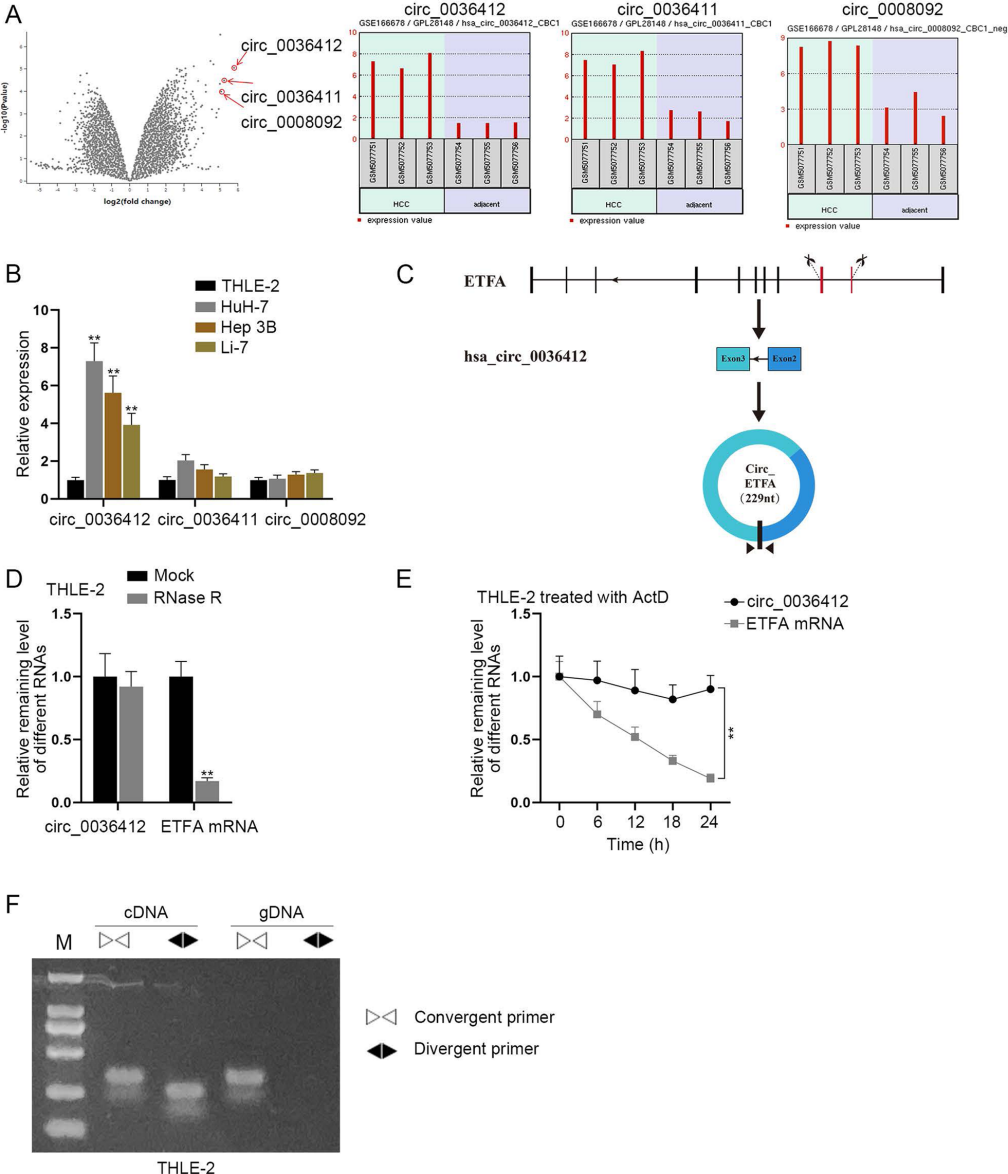
The characterization of circ_0036412 in HCC cells. **A** GSE113510 dataset was adopted to analyze the circRNAs differentially expressed in HCC tissues compared with normal tissues. **B** The expressions of circ_0036412, circ_0036411 and circ_0008092 were detected by qRT-PCR in THLE-2, Hep 3B, HuH-7 and Li-7 cells (One-way ANOVA, Tukey). **C** The circular structure of circ_0036412 was shown. **D** The expressions of circ_0036412 and ETFA mRNA were detected by qRT-PCR in THLE-2 cells after the treatment of RNase R (Student’s t test). **E** The expressions of circ_0036412 and ETFA mRNA were detected by qRT-PCR in ActD-treated THLE-2 cells (Student’s t test). **F** PCR-agarose gel electrophoresis in THLE-2 cells verified the circular structure of circ_0036412. **P < 0.01

Gene Expression Ominibus (GEO) database (https://www.ncbi.nlm.nih.gov/geo/) was firstly adopted to screen differentially expressed circRNAs in HCC tissues and adjacent tissues. According to the logFC descending order, we selected the top three circRNAs, circ_0036412, circ_0036411 and circ_0008092 as shown in GSE113510 dataset (Fig. 1A). There were no reports on these candidates in HCC. Next, we implemented qRT-PCR to detect the expressions of these candidates in HCC cell lines (Hep 3B, HuH-7 and Li-7) and normal cell line (THLE-2). The results showcased that, circ_0036412 expression was most up-regulated in HCC cell lines in comparison with that in THLE-2 cells, especially in HuH-7 and Hep 3B cells (Fig. 1B). Therefore, we chose circ_0036412, and HuH-7 and Hep 3B cells for follow-up experiments. As shown in Fig. 1C, circ_0036412, with its host gene as ETFA, features circular structure. Afterwards, qRT-PCR analyses were performed in THLE-2 cells to assess the stability of circ_0036412. As shown in Fig. 1D, circ_0036412 expression remained almost unchanged, while ETFA mRNA was nearly completely digested by RNase R. Moreover, we performed qRT-PCR to detect the expressions of circ_0036412 and ETFA mRNA at 0, 6, 12, 18, and 24 h, respectively in THLE-2 cells treated by ActD, a transcription inhibitor. As indicated in Fig. 1E, ETFA mRNA degraded at a faster rate than circ_0036412. The results of qRT-PCR proved the circular structure of circ_0036412, as circRNA is more stable than linear RNA. To further verify its circular structure, PCR-agarose gel electrophoresis was performed in THLE-2 cells. It was unmasked by the results that circ_0036412 was amplified in cDNA by both convergent primer and divergent primer, while it was amplified in genomic DNA (gDNA) only by convergent primer (Fig. 1F). Taken together, circ_0036412 is overexpressed in HCC cells and features circular structure.

### PRDM1 activates circ_0036412 transcription to facilitate the proliferation and inhibit the cell cycle arrest of HCC cells in vitro

**Fig. 2:**
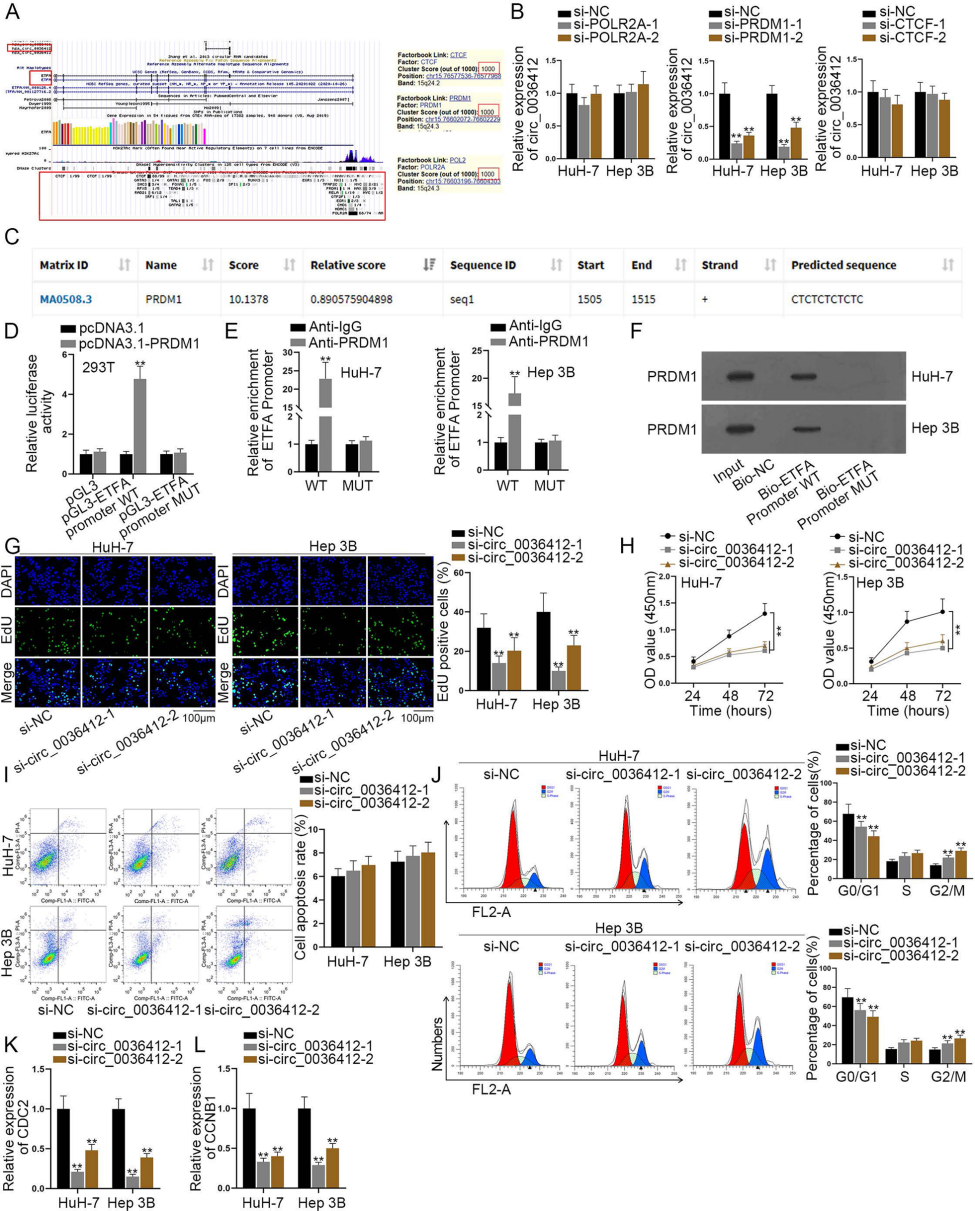
PRDM1 activates circ_0036412 transcription to facilitate the proliferation and inhibit the cell cycle of HCC cells in vitro. **A** UCSC (http://genome-asia.ucsc.edu/index.html) was used to predict the transcription factors regulating ETFA. **B** The expression of circ_0036412 was detected by qRT-PCR in HuH-7 and Hep 3B cells after the transfection of si-CTCF-1/2, si-PRDM1-1/2 or si-POLR2A-1/2 (One-way ANOVA, Dunnett). **C** JASPAR (https://jaspar.genereg.net/) predicted the binding sites of PRDM1 and ETFA promoter sequences. The site was located on the 1505–1515 fragment, and the sequence is CTCTCTCTCTC. **D** Luciferase reporter assay detected the interaction between PRDM1 and ETFA promoter in 293 T cells (Two-way ANOVA, Tukey). **E**–**F** ChIP (Student’s test) and DNA pulldown assays detected the interaction between PRDM1 and ETFA promoter in HuH-7 and Hep 3B cells. **G**, **H** EdU and CCK-8 assays evaluated cell proliferation in HuH-7 and Hep 3B cells after the transfection of si-circ_0036412-1/2 (One-way ANOVA, Dunnett). (I) TUNEL assay assessed cell apoptosis in HuH-7 and Hep 3B cells after the transfection of si-circ_0036412-1/2 (One-way ANOVA, Dunnett). **J** Flow cytometry analysis testified cell cycle in HuH-7 and Hep 3B cells after the transfection of si-circ_0036412-1/2 (One-way ANOVA, Dunnett). **K**, **L** The mRNA levels of CDC2 and CCNB1 were detected by qRT-PCR in HuH-7 and Hep 3B cells after the transfection of si-circ_0036412-1/2 (One-way ANOVA, Dunnett). **P < 0.01

According to the results of Fig. 1A, B, circ_0036412 expression was proved to be significantly up-regulated in HCC tissues and cells. We speculated that ETFA transcription might be activated by a transcription factor to enhance the expression of circ_0036412. Therefore, we firstly explored the transcription factors that can regulate circ_0036412. We used UCSC (http://genome-asia.ucsc.edu/index.html) to search the possible transcription factors regulating ETFA. Three transcription factors with the highest scores, namely, CTCF, PRDM1 and POLR2A were chosen (Fig. 2A). We then conducted qRT-PCR to detect the knockdown efficiency of si-PRDM1-1/2/3, si-POLR2A-1/2/3 and si-CTCF-1/2/3, respectively in HuH-7 and Hep 3B cells. It was shown that their expressions were down-regulated by the knockdown vectors compared with si-NC (Additional file 1: Fig. S1A–C). Due to the higher efficiency, we selected si-PRDM1-1/2, si-POLR2A-1/2 and si-CTCF-1/2 for follow-up experiments. Subsequently, we conducted qRT-PCR in HCC cells to detect circ_0036412 expression using si-PRDM1-1/2, si-POLR2A-1/2 and si-CTCF-1/2, respectively. The results showed that circ_0036412 expression was decreased most significantly after interference with PRDM1 (Fig. 2B). Hence, we selected PRDM1 for further study. Afterwards, JASPAR (https://jaspar.genereg.net/) was used to predict the binding sites of PRDM1 and ETFA promoter sequences. We selected the site with the highest score (Fig. 2C). We then conducted qRT-PCR in 293 T cells to detect the overexpression efficiency of pcDNA3.1-PRDM1. It was shown that PRDM1 was overexpressed by pcDNA3.1-PRDM1 (Additional file 1: Fig. S1D). Next, we performed luciferase reporter assay to detect the interaction between PRDM1 and ETFA promoter in 293 T cells. It was demonstrated by the results that the luciferase activity was increased after the overexpression of PRDM1 in pGL3-ETFA promoter WT while the luciferase activity remained unchanged in pGL3-ETFA promoter MUT, compared with the control group. This indicated that PRDM1 can transcribe and activate the promoter region of ETFA (Fig. 2D). ChIP, followed by qRT-PCR, detected the binding of PRDM1 to ETFA promoter. It was shown that ETFA promoter was preferentially enriched in the products precipitated by Anti-PRDM1. The results suggested the interaction between PRDM1 and ETFA promoter (Fig. 2E). In addition, DNA pulldown experiment, followed by western blot, was implemented to further prove the interaction between PRDM1 and ETFA promoter. It was found that PRDM1 was preferentially enriched in biotinylated ETFA promoter group while the enrichment remained steady after the mutation compared with the control group (Fig. 2F). We implemented qRT-PCR to detect the knockdown efficiency of si-circ_0036412-1/2/3 in HuH-7 and Hep 3B cells. It was found that circ_0036412 expression was attenuated by the knockdown vectors (Additional file 1: Fig. S1E). Because of the higher efficiency, si-circ_0036412-1 and si-circ_0036412-2 were chosen for follow-up experiments. Next, we performed experiments to explore the biological functions of circ_0036412 in HCC cells. We conducted EdU and CCK-8 assays to detect HCC cell proliferation in HCC cells after the transfection of si-circ_0036412-1/2. The results showed that circ_0036412 ablation led to the decrease in EdU positive cells and OD value, indicating the suppression of cell proliferation (Fig. 2G, H). We then performed TUNEL assay in HCC cells to evaluate HCC cell apoptosis after the transfection of si-circ_0036412-1/2. As shown in Fig. 2I, cell apoptosis rate remained stable after interference with circ_0036412, indicating that circ_0036412 cannot affect HCC cell apoptosis. We next implemented flow cytometry analysis to detect HCC cell cycle in HCC cells after circ_0036412 knockdown. The results showed that circ_0036412 depletion could induce G2/M phase arrest of HCC cells (Fig. 2J). For further validation, qRT-PCR was conducted to detect cell cycle-related genes (CDC2 and CCNB1) in HCC cells after circ_0036412 silencing. The results showed that the mRNA levels of CDC2 and CCNB1 were down-regulated after circ_0036412 depletion, indicating that circ_0036412 ablation can induce G2/M phase arrest of HCC cells (Fig. 2K, L). Taken together, PRDM1 activates circ_0036412 transcription to facilitate the proliferation and inhibit the cell cycle arrest of HCC cells in vitro.

### Circ_0036412 modulates Hedgehog signaling pathway in HCC cells

**Fig. 3:**
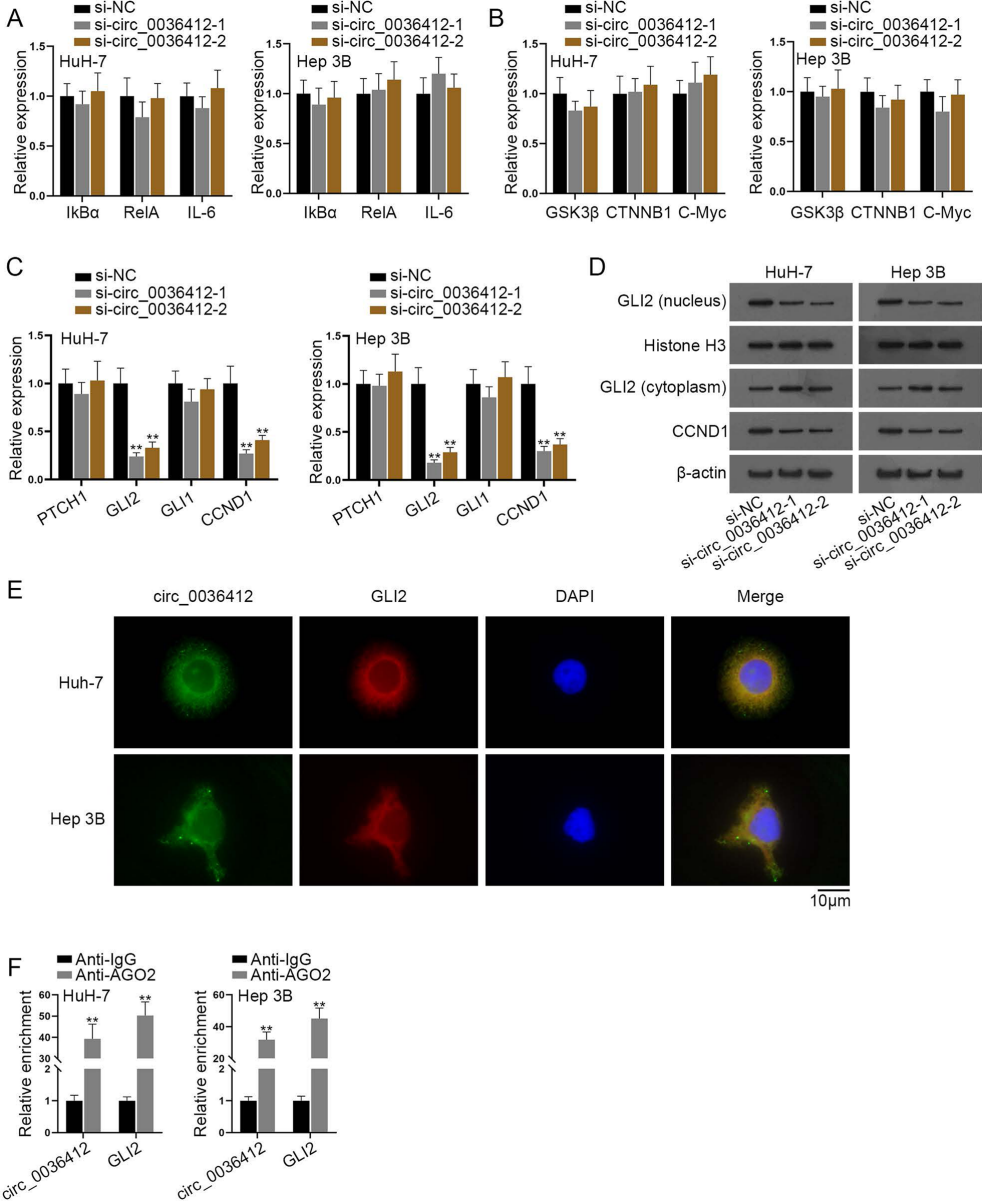
Circ_0036412 modulates Hedgehog signaling pathway in HCC cells. **A**–**C** The expressions of IkBα, RelA, IL-6, GSK3β, CTNNB1, C-Myc, PTCH1, GLI2 and CCND1 were detected by qRT-PCR in HuH-7 and Hep 3B cells after the transfection of si-circ_0036412-1/2 (One-way ANOVA, Dunnett). **D** The protein levels of nuclear GLI2, cytoplasmic GLI2 and CCND1 were detected by western blot in HuH-7 and Hep 3B cells after the transfection of si-circ_0036412-1/2. **E** FISH assay detected the co-localization of circ_0036412 and GLI2 in HuH-7 and Hep 3B cells. **F** RIP assay detected the existence of circ_0036412 and GLI2 in RISC in HuH-7 and Hep 3B cells (Student’s t test). **P < 0.01

The results in Fig. 2 showed that circ_0036412 promotes proliferation and inhibits G2/M phase arrest in vitro, but the specific regulatory mechanism still remained unclear. According to the literature review, Hedgehog [23], NF-kB [24] and WNT [25] signaling pathways have been reported to facilitate the development of HCC. Hence, we next explored whether circ_0036412 regulates signaling pathways in HCC cells. Firstly, qRT-PCR was implemented to detect the expressions of target genes of NF-kB pathway (IkBα, RelA and IL-6) in HuH-7 and Hep 3B cells after the transfection of si-circ_0036412-1/2. It was unearthed by the results that interference with circ_0036412 did not affect the activity of NF-kB pathway (Fig. 3A). Afterwards, qRT-PCR was implemented to detect the expressions of target genes of WNT pathway (GSK3β, CTNNB1 and C-Myc) in HCC cells after the transfection of si-circ_0036412-1/2. It was shown by the results that circ_0036412 knockdown did not affect the activity of WNT pathway (Fig. 3B). We then conducted qRT-PCR to detect the expressions of target genes of Hedgehog pathway (PTCH1, GLI1, GLI2 and CCND1) in HCC cells after the transfection of si-circ_0036412-1/2. It was unmasked by the results that circ_0036412 ablation reduced the expressions of GLI2 and its downstream target CCND1, indicating that circ_0036412 might positively regulate GLI2 to improve the activity of Hedgehog pathway (Fig. 3C). Subsequently, we performed western blot to evaluate the protein levels of GLI2 and CCND1 after the transfection of si-circ_0036412-1/2. The results showed that after interference with circ_0036412, the protein levels of nuclear GLI2 and CCND1were decreased while that of cytoplasmic GLI2 was increased, further indicating that circ_0036412 interference can reduce the activity of Hedgehog pathway (Fig. 3D). FISH was performed to detect the co-localization of circ_0036412 and GLI2 in HCC cells. The results showed that circ_0036412 and GLI2 were co-localized in HCC cells (Fig. 3E). RIP assay, followed by qRT-PCR, was implemented to evaluate the enrichments of circ_0036412 and GLI2 in the products precipitated by Anti-IgG and Anti-AGO2. The results showed the preferential enrichments of circ_0036412 and GLI2 in the products precipitated by Anti-AGO2, suggesting the existence of circ_0036412 and GLI2 in RNA-induced silencing complex (RISC) as AGO2 is the major component of RISC (Fig. 3F). To sum up, circ_0036412 modulates Hedgehog signaling pathway in HCC cells.

### GLI2 propels HCC growth in vivo

**Fig. 4:**
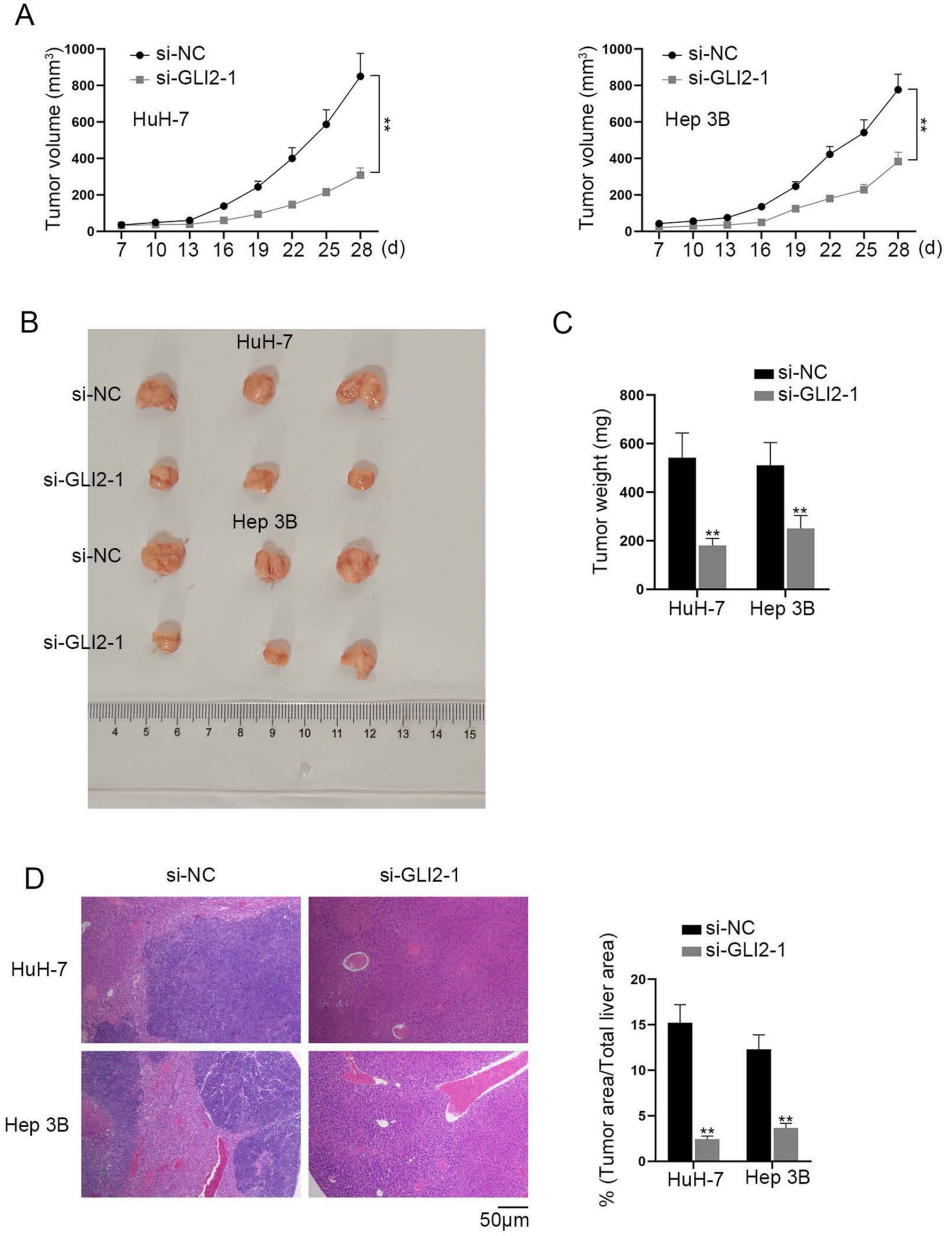
GLI2 propels HCC growth in vivo. **A** Tumor volume was detected every 3 days after the injection of HuH-7 and Hep 3B cells transfected with si-NC or si-GLI2-1 into the nude mice (Student’s t test). **B** Tumor weight was measured 28 days after the injection (Student’s t test). **C** HE staining was used to calculate tumor area/total liver area in the stained liver tissues (Student’s t test). **P < 0.01. **D** H&E staining of liver tissues extracted from different group of mice was used to detect liver metastasis

It has been reported that GLI2 have oncogenic potential in HCC in vivo [26]. Hence, we next explored the effects of GLI2 on HCC in vivo. Firstly, we used qRT-PCR to detect GLI2 expression after the transfection of si-GLI2-1/2/3 into HuH-7 and Hep 3B cells. It was shown that its expression was down-regulated by the knockdown vectors (Additional file 1: Fig. S1F). Due to the higher efficiency, si-GLI2-1 was chosen for follow-up experiments. HCC cells were subcutaneously injected into the mice 48 h after the transfection of si-GLI2-1. We measured tumor volume every three days, finding that tumor growth was inhibited in si-GLI2-1 group compared to that in the control group (Fig. 4A). Twenty-eight days after the injection, we resected the tumors from the mice and measured tumor volume and weight. As shown in Fig. 4B, tumor volume was inhibited in si-GLI2-1 group compared with the control group. Furthermore, it was found that GLI2 ablation suppressed the tumor weight in si-GLI2-1 group compared with the control group (Fig. 4C). The results of H&E staining showed that silencing of GLI2 suppressed liver metastasis (Fig. 4D). Taken together, GLI2 propels HCC growth in vivo.

### Circ_0036412 up-regulates GLI2 expression by competitively binding to miR-579-3p to regulate the proliferation and cell cycle of HCC cells

**Fig. 5:**
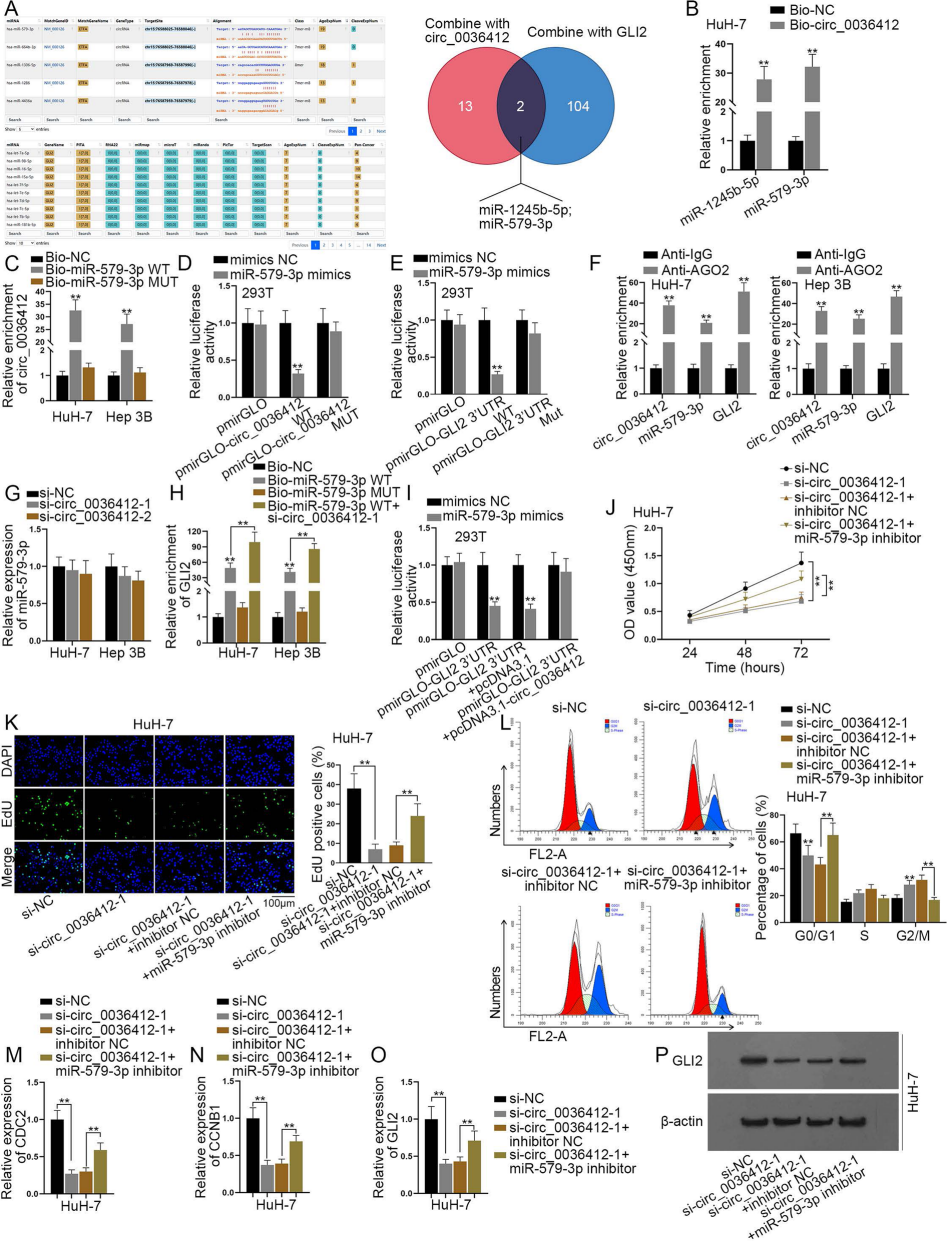
Circ_0036412 up-regulates GLI2 expression by competitively binding to miR-579-3p to regulate the proliferation and cell cycle of HCC cells. **A** StarBase predicted the miRNAs interacting with circ_0036412 and GLI2. The Venn diagram showed the common miRNAs both binding to circ_0036412 and GLI2. **B** RNA pulldown assay followed by qRT-PCR in HuH-7 and Hep 3B cells detected the enrichments of miR-1245b-5p and miR-579-3p in Bio-circ_0036412 group compared with control group (Student’s t test). **C** RNA pulldown assay followed by qRT-PCR in HuH-7 and Hep 3B cells evaluated the combination between miR-579-3p and circ_0036412 (One-way ANOVA, Dunnett). **D**, **E** Luciferase reporter assays were conducted to testify the interaction between circ_0036412 and GLI2 3′UTR in 293 T cells (Two-way ANOVA, Tukey). **F** RIP assay followed by qRT-PCR in HuH-7 and Hep 3B cells detected the existence of miR-579-3p and circ_0036412 and GLI2 in RISC (Student’s t test). **G** The expression of miR-579-3p was detected by qRT-PCR in HuH-7 and Hep 3B cells after circ_0036412 knockdown (One-way ANOVA, Dunnett). **H** RNA pulldown assay in HuH-7 and Hep 3B cells detected the enrichment of GLI2 in Bio-NC, Bio-miR-579-3p WT, Bio-miR-579-3p MUT and Bio-miR-579-3p WT + si-circ_0036412-1 groups (One-way ANOVA, Tukey). **I** Luciferase reporter assay assessed the luciferase activity of pmirGLO, pmirGLO-GLI2 3′UTR, pmirGLO-GLI2 3′UTR + pcDNA3.1 and pmirGLO-GLI2 3′UTR + pcDNA3.1-circ_0036412 after the co-transfection with mimics NC or mir-579-3p mimics into 293 T cells (Two-way ANOVA, Tukey). **J**–**K** CCK-8 and EdU assays evaluated cell proliferation in HuH-7 cells after the transfection of si-NC, si-circ_0036412-1, si-circ_0036412-1 + inhibitor NC or si-circ_0036412-1 + miR-579-3p inhibitor (One-way ANOVA, Tukey). **L** Flow cytometry analysis testified cell cycle in HuH-7 cells after the transfection of si-NC, si-circ_0036412-1, si-circ_0036412-1 + inhibitor NC or si-circ_0036412-1 + miR-579-3p inhibitor (One-way ANOVA, Tukey). **M**, **N** The mRNA levels of CDC2 and CCNB1 were detected by qRT-PCR in HuH-7 cells after the transfection of si-NC, si-circ_0036412-1, si-circ_0036412-1 + inhibitor NC or si-circ_0036412-1 + miR-579-3p inhibitor (One-way ANOVA, Tukey). **O**–**P** The mRNA and protein levels of GLI2 were detected by qRT-PCR (One-way ANOVA, Tukey) and western blot in HuH-7 cells after the transfection of si-NC, si-circ_0036412-1, si-circ_0036412-1 + inhibitor NC or si-circ_0036412-1 + miR-579-3p inhibitor. **P < 0.01

As indicated by the results of RIP assay in Fig. 3F, we speculated that circ_0036412 may regulate GLI2 through ceRNA mode. We utilized starBase to predict the miRNAs interacting with circ_0036412 and GLI2, respectively. Afterwards, Venny 2.1 (https://bioinfogp.cnb.csic.es/tools/venny/index.html) was used for intersection analysis. As shown in the Venn diagram, we obtained the common miRNAs both binding to circ_0036412 and GLI2, namely, miR-1245b-5p and miR-579-3p (Fig. 5A). As indicated in the previous studies, miR-1245b-5p serves as a suppressor in prostate cancer cell [27]; and miR-579-3p inhibits the malignant progression of squamous cell lung cancer [28]. To identify the most relevant miRNA, we performed RNA pulldown assay followed by qRT-PCR in HuH-7 cells and discovered that miR-579-3p was most enriched in Bio-circ_0036412 group compared with miR-1245-5p (Fig. 5B). Therefore, we chose miR-579-3p for follow-up experiments. RNA pulldown assay followed by qRT-PCR was performed to detect the interaction between miR-579-3p and circ_0036412. It was shown that circ_0036412 was most enriched in Bio-miR-579-3p WT group compared with Bio-NC group, while circ_0036412 enrichment had no remarkable change in Bio-miR-579-3p MUT group. The results suggested the interaction between circ_0036412 and miR-579-3p (Fig. 5C). Luciferase reporter assays were performed in 293 T cells, showing that miR-579-3p mimics suppressed the luciferase activity in pmirGLO-circ_0036412 WT and pmirGLO-GLI2 3’UTR WT groups instead of that in the mutant groups, compared with control groups. The results suggested the interaction between miR-579-3p and circ_0036412, and miR-579-3p and GLI2 3′UTR (Fig. 5D, E). Furthermore, RIP assay followed by qRT-PCR was performed in HuH-7 and Hep 3B cells, showcasing that circ_0036412, miR-579-3p and GLI2 were preferentially enriched in Anti-AGO2 group compared to that in Anti-IgG group. The results indicated the coexistence of circ_0036412, miR-579-3p and GLI2 in RISC (Fig. 5F). Afterwards, we implemented qRT-PCR to testify miR-579-3p expression after circ_0036412 knockdown in HCC cells. It was unearthed by the results that circ_0036412 depletion could not affect miR-579-3p expression (Fig. 5G). We then implemented RNA pulldown assay followed by qRT-PCR, discovering that GLI2 was preferentially enriched in Bio-miR-579-3p WT group while GLI2 expression couldn’t be up-regulated in Bio-miR-579-3p MUT group compared with Bio-NC. In addition, the transfection of si-circ_0036412-1 enhanced the enrichment in Bio-miR-579-3p WT group. The results suggested that GLI2 can bind to miR-579-3p, and the interaction is enhanced after circ_0036412 interference (Fig. 5H). We performed qRT-PCR to detect the overexpression efficiency of pcDNA3.1-circ_0036412 in 293 T and HuH-7 cells. It was found that the expression of circ_0036412 was up-regulated by the overexpression vector (Additional file 1: Fig. S1G). We then performed luciferase reporter assay, discovering that the luciferase activity of pmirGLO-GLI2 3’UTR group was decreased by miR-579-3p mimics, and was then completely reversed by overexpression of circ_0036412 (Fig. 5I). Next, we performed rescue experiments. Functional experiments, qRT-PCR and western blot were performed after the transfection of si-NC, si-circ_0036412-1, si-circ_0036412-1 + inhibitor NC or si-circ_0036412-1 + miR-579-3p inhibitor. We conducted CCK-8 and EdU assays in HuH-7 cells to evaluate cell proliferation. It was found that HCC cell proliferation was reduced by circ_0036412 ablation, and was then partially reversed by miR-579-3p inhibition (Fig. 5J, K). We next conducted flow cytometry analysis to detect HCC cell cycle in HuH-7 cells. It was shown that G2/M phase arrest was induced by circ_0036412 ablation, and was then partially reversed by miR-579-3p inhibition (Fig. 5L). To further verify the effects on cell cycle, qRT-PCR was performed to detect the expressions of CDC2 and CCNB1 in HuH-7 cells. It was shown that the mRNA levels of CDC2 and CCNB1 were reduced after interference with circ_0036412, and were then partially reversed by miR-579-3p inhibition (Fig. 5M, N). In addition, qRT-PCR and western blot were performed to detect circ_0036412/miR-579-3p/GLI2. It was shown that GLI2 expression was reduced by circ_0036412 ablation, and was then partially reversed by miR-579-3p inhibition (Fig. 5O, P). Taken together, circ_0036412 up-regulates GLI2 expression by competitively binding to miR-579-3p to regulate the proliferation and cell cycle of HCC cells.

### Circ_0036412 stabilizes GLI2 expression by recruiting ELAVL1

**Fig. 6:**
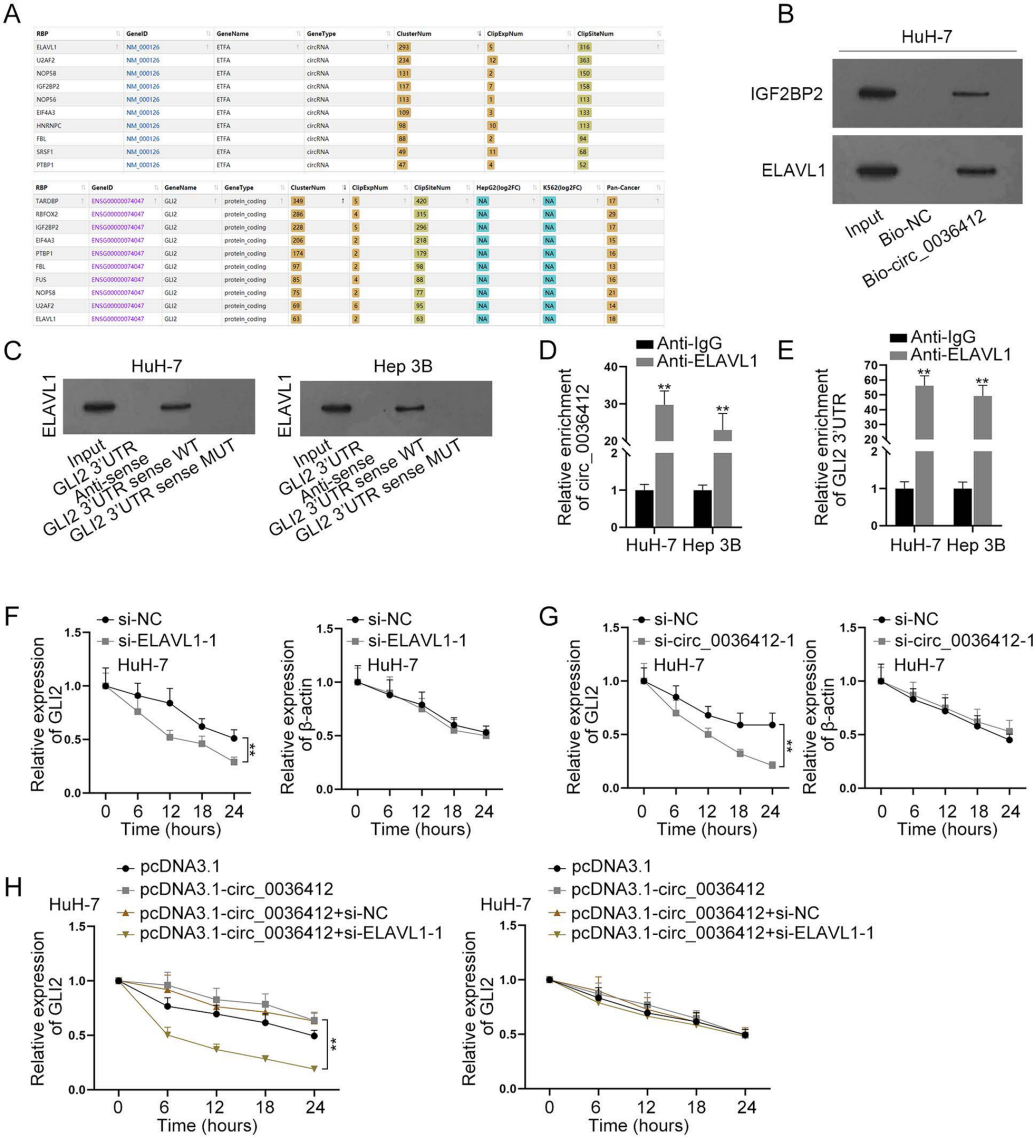
Circ_0036412 stabilizes GLI2 expression by recruiting ELAVL1. **A** StarBase predicted the RBPs interacting with circ_0036412 and GLI2. **B** RNA pulldown assay followed by western blot detected the interaction of circ_0036412 with IGF2BP2 or ELAVL1 in HuH-7 cells. **C** RNA pulldown assay followed by western blot detected the interaction of circ_0036412 with ELAVL1 in HuH-7 and Hep 3B cells. **D**, **E** RIP assay followed by qRT-PCR assessed the interaction of ELAVL1 with circ_0036412 and GLI2 3’UTR in HuH-7 and Hep 3B cells (Student’s t test). **F**, **G** The half-lives of GLI2 and β-actin were assessed by qRT-PCR in α-amanitin-treated HuH-7 cells after the knockdown of ELAVL1 or circ_0036412 (Student’s t test). **H** The half-lives of GLI2 and β-actin were assessed by qRT-PCR in α-amanitin-treated HuH-7 cells after the transfection of pcDNA3.1, pcDNA3.1-circ_0036412, pcDNA3.1-circ_0036412 + si-NC or pcDNA3.1-circ_0036412 + si-ELAVL1-1 (One-way ANOVA, Tukey). **P < 0.01

According to the experimental results in Fig. 5, we found that the silenced miR-579-3p could only partially reverse the effects of down-regulated circ_0036412 expression on HCC cell progression and GLI2 expression. Hence, circ_0036412 may also regulate GLI2 through other pathways. Due to the fact that circ_0036412 is mainly located in cytoplasm, we conjectured that circ_0036412 may regulate GLI2 by recruiting RNA-binding proteins (RBPs), which is post-transcriptional regulation. We used starBase database for predicting the RBPs interacting with circ_0036412 and GLI2. According to the descending order of ClusterNum, we acquired the top ten candidates binding to circ_0036412 and top ten candidates binding to GLI2. Seven common ones interacting with both were selected, namely, U2AF2, EIF4A3, NOP58, FBL, IGF2BP2, ELAVL1 and PTBP1 (Fig. 6A). Human Protein Atlas (www.proteinatlas.org) was used to predict the subcellular location of these RBPs (Additional file 2: Fig. S2A–G). Since circ_0036412 is mainly located in the cytoplasm, we selected ELAVL1 and IGF2BP2, which is also located in the cytoplasm. Afterwards, we performed RNA pulldown assay followed by western blot in HuH-7 cells to assess the enrichments of ELAVL1 and IGF2BP2 in Bio-circ_0036412 group compared with Bio-NC. It was found that the enrichment of ELAVL1 was higher than that of IGF2BP2 (Fig. 6B). Furthermore, it has been reported that ELAVL1 can stabilize downstream mRNAs [29]. Based on the reference and the experimental results, we selected ELAVL1 for the follow-up experiments. We performed RNA pulldown assay followed by western blot in HuH-7 and Hep 3B cells to detect the interaction between GLI2 3’UTR and ELAVL1. The results showed that ELAVL1 was preferentially pulled down by GLI2 3’UTR in GLI2 3’UTR group, proving the interaction. However, the interaction in GLI2 3′UTR Anti-sense and GLI2 3’UTR sense MUT groups was disrupted (Fig. 6C). Next, RIP followed by qRT-PCR in HCC cells indicated the interaction of ELAVL1 with circ_0036412 and GLI2 3’UTR (Fig. 6D, E). We then performed qRT-PCR in HCC cells to detect the knockdown efficiency of si-ELAVL1-1/2/3. It’s unearthed by the results that these knockdown vectors inhibited ELAVL1 expression (Additional file 2: Fig. S2H). Due to the higher efficiency, we selected si-ELAVL1-1 for the follow-up assays. Subsequently, we performed qRT-PCR to detect the half-life of GLI2 and β-actin in α-amanitin-treated HuH-7 cells after the knockdown of ELAVL1 or circ_0036412. It was shown that the depletion of ELAVL1 or circ_0036412 reduced the half-life and stability of GLI2 versus that of the control group (Fig. 6F, G). To investigate the role of circ_0036412/ELAVL1 axis in regulating GLI2 stability, we performed qRT-PCR to detect the half-life GLI2 and β-actin in α-amanitin-treated HuH-7 cells after the transfection of pcDNA3.1, pcDNA3.1-circ_0036412, pcDNA3.1-circ_0036412 + si-NC or pcDNA3.1-circ_0036412 + si-ELAVL1-1. We found that the half-life of GLI2 was increased by circ_0036412 overexpression, and was then reduced to a lower level than normal by ELAVL1 inhibition. The results indicated that the stability of GLI2 must be adjusted by ELAVL1 (Fig. 6H). Taken together, circ_0036412 stabilizes GLI2 expression by recruiting ELAVL1.

### Circ_0036412 promotes the proliferation and inhibits cell cycle arrest of HCC cells in vitro through Hedgehog pathway

**Fig. 7:**
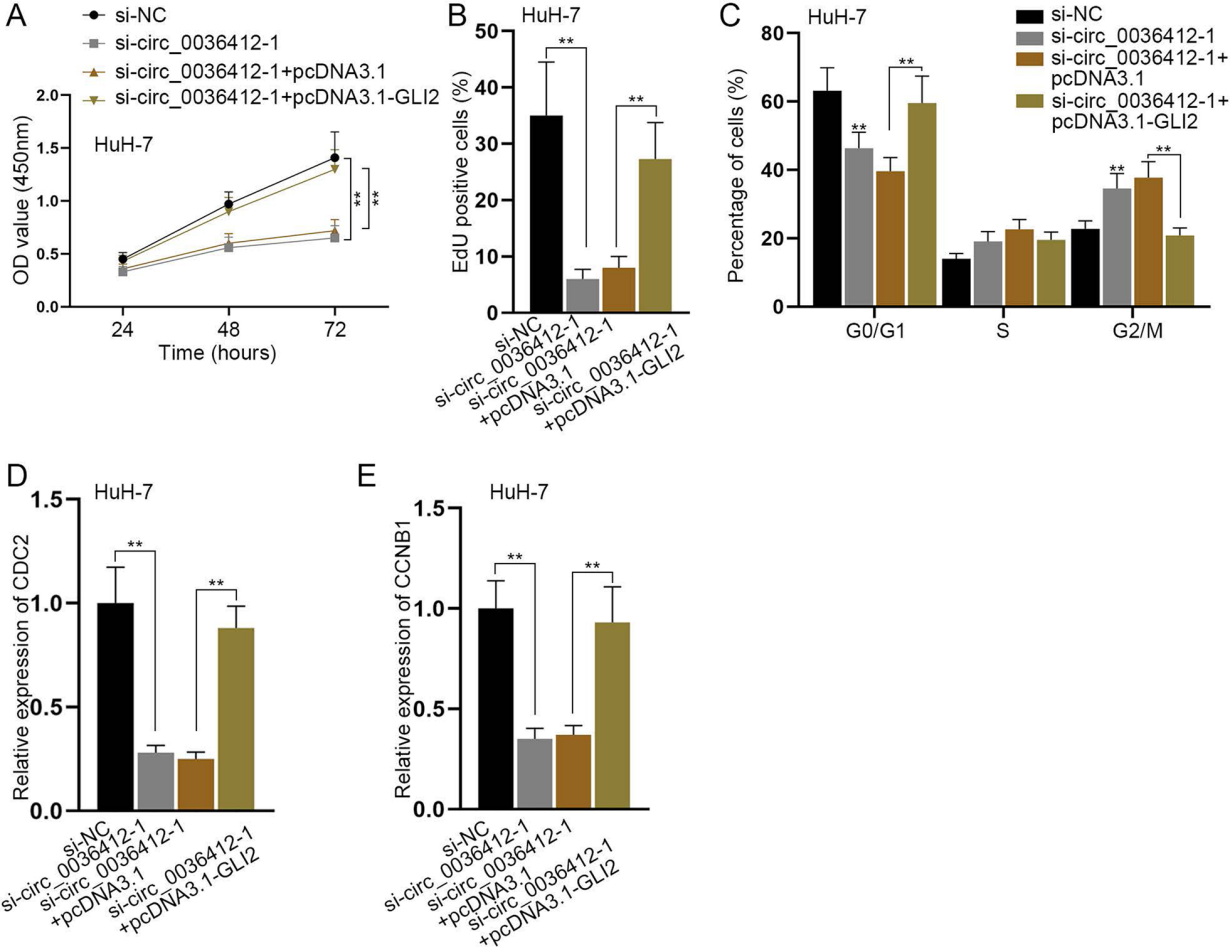
Circ_0036412 propels the proliferation and suppresses cell cycle arrest of HCC cells in vitro through Hedgehog pathway. **A**, **B** CCK-8 and EdU assays assessed cell proliferation in HuH-7 cells after the transfection of si-NC, si-circ_0036412-1, si-circ_0036412-1 + pcDNA3.1 or si-circ_0036412-1 + pcDNA3.1-GLI2 (One-way ANOVA, Tukey). **C** Flow cytometry analysis detected cell cycle in HuH-7 cells after the transfection of si-NC, si-circ_0036412-1, si-circ_0036412-1 + pcDNA3.1 or si-circ_0036412-1 + pcDNA3.1-GLI2 (One-way ANOVA, Tukey). **D**, **E** The mRNA levels of CDC2 and CCNB1 were evaluated by qRT-PCR in HuH-7 cells after the transfection of si-NC, si-circ_0036412-1, si-circ_0036412-1 + pcDNA3.1 or si-circ_0036412-1 + pcDNA3.1-GLI2 (One-way ANOVA, Tukey). *P < 0.05, **P < 0.01

The results in Fig. 2 indicated that circ_0036412 promotes HCC cell proliferation and inhibits G2/M phase arrest, and the results of Figs. 3, 4, 5 and 6 confirmed that circ_0036412 promotes the expression of GLI2 through different regulatory methods. Thus, we next explored whether circ_0036412 can regulate HCC cell progression via GLI2 in vitro. Firstly, we performed qRT-PCR to detect the overexpression efficiency of pcDNA3.1-GLI2 in HuH-7 cells. It was found that GLI2 expression was reinforced by the overexpression vector (Additional file 2: Fig. S2I). Subsequently, we performed rescue experiments. Functional experiments and qRT-PCR were conducted in HuH-7 cells after the transfection of si-NC, si-circ_0036412-1, si-circ_0036412-1 + pcDNA3.1 or si-circ_0036412-1 + pcDNA3.1-GLI2. The results of CCK-8 and EdU assays demonstrated that HCC cell proliferation was reduced by circ_0036412 ablation, and was then completely countervailed by GLI2 overexpression (Fig. 7A, B). The results of flow cytometry analysis showed that G2/M phase arrest was promoted by circ_0036412 depletion, and was then fully reversed by GLI2 overexpression (Fig. 7C). Furthermore, the results of qRT-PCR showed that the mRNA levels of CDC2 and CCNB1 were decreased after interference with circ_0036412, and were then fully counteracted by GLI2 overexpression (Fig. 7D, E). The above results indicated that circ_0036412 affects the proliferation and cell cycle of HCC cells in vitro via GLI2. As GLI2 is the target gene of Hedgehog pathway, we concluded that circ_0036412 facilitates the proliferation and inhibits cell cycle arrest of HCC cells in vitro through Hedgehog pathway.

## Discussion

As the most common class of primary liver cancer, HCC features high mortality rate [1]. The overall survival and prognosis of HCC patients is poor owing to its recurrence and metastasis, which are difficulty to be predicted [6, 7]. This led to our study intending to explore the underlying mechanisms and potential biomarkers of HCC.

We used GEO database and qRT-PCR to identify the most relevant circRNA, namely, circ_0036412. Afterwards, the circular structure of circ_0036412 was proved by PCR-agarose gel electrophoresis and qRT-PCR. Based on the literature review, we found that circFBXO11 regulates HCC cell proliferation and cell cycle [30] and circSOD2 modulates in vitro HCC cell proliferation and cell cycle [31], verifying the role of circRNAs in HCC. In our study, we performed CCK-8, EdU, TUNEL, flow cytometry and qRT-PCR to prove that circ_0036412 facilitates the proliferation and inhibits the G2/M phase arrest of HCC cells in vitro. Circ_0036412 has been reported to promote HCC progression by sequestering miR-612 and recruiting EIF4A3 [13], which is in line with the abovementioned results. Although the oncogenic role of circ_0036412 was clarified by previous study and our study, the mechanisms by which circ_0036412 promotes HCC progression are different.

According to literature review, PRDM1 was reported to be overexpressed in HCC [32]. We used bioinformatics and qRT-PCR to identify PRDM1 as the transcriptional factor of circ_0036412. The results of luciferase reporter, ChIP and DNA pulldown assays verified the interaction between PRDM1 and circ_0036412. The above results proved that PRDM1 can transcriptionally activate circ_0036412 in HCC cells.

In accordance with the literature review, Hedgehog [23], NF-kB [24] and WNT [25] signaling pathways have been reported to facilitate the development of HCC. We performed qRT-PCR to exclude the possibility of NF-kB and WNT signaling pathways. The results of western blot showcased the effect of circ_0036412 depletion on the target genes of Hedgehog pathway (GLI2 and CCND1), proving that circ_0036412 can modulate Hedgehog pathway in HCC cells. It has been reported that GLI2 ablation inhibits xenograft growth of cervical cancer cells in vivo [33]. We performed animal experiments and found that GLI2 propels HCC growth in vivo. Afterwards, in our study, we proved that circ_0036412 affects the proliferation and cell cycle of HCC cells in vitro via GLI2. The above results suggested that circ_0036412 facilitates the proliferation and cell cycle of HCC cells through Hedgehog pathway, which is in line with the findings in the previous study.

RBP has been reported to modulate HCC progression by interacting with the target mRNA [34]. Through bioinformatics and RNA pulldown assay, we identified ELAVL1 as the RBP for circ_0036412. It has been reported that ELAVL1 mediates the stability of BECN1 mRNA in hepatic stellate cells [29]. Moreover, ELAVL1 directly stabilizes LINC00336 in lung cancer cells [35]. We used mechanism experiments to demonstrate that ELAVL1 can interact with circ_0036412 and GLI2 3’UTR. The results of qRT-PCR verified that circ_0036412 improves the stability of GLI2 via ELAVL1. The above results indicated that circ_0036412 stabilizes GLI2 expression by recruiting ELAVL1.

In the current study, it was speculated that circ_0036412 exert its regulatory role in HCC cells via ceRNA mode. CircRNAs have been widely reported to function as ceRNAs in HCC. Circ_0001955 promotes HCC cell progression through sponging miR-516a-5p to enhance the expressions of TRAF6 and MAPK11 [36]; circ_102559 sequesters miR-130a-5p to propel HCC cell progression by up-regulating ANXA2 expression [37]; and circ_0091570 serves as a ceRNA to inhibit the progression of HCC via sequestering miR-1307 [38]. We used bioinformatics to screen out the miRNAs both interacting with circ_0036412 and GLI2, followed by RNA pulldown assay to identify miR-579-3p. Mechanism experiments were implemented to prove the interaction of miR-579-3p with circ_0036412 and GLI2. Rescue experiments explored the mechanisms among circ_0036412, miR-579-3p and GLI2. The above results indicated that circ_0036412 up-regulates GLI2 expression by sponging miR-579-3p to regulate the proliferation and cell cycle of HCC cells.

## Conclusion

In conclusion, circ_0036412 promotes the proliferation and inhibits G2/M phase arrest of HCC cells via Hedgehog signaling pathway. In our study, we probed into the roles of circ_0036412/miR-579-3p/GLI2 regulatory axis and Hedgehog pathway in HCC. Our study firstly validated that circ_0036412 regulates GLI2 via ceRNA mode and the recruitment of ELAVL1. Furthermore, it was firstly proved that circ_0036412 can affect proliferation and cell cycle of HCC cells. The present study explored the potential of circ_0036412 as a biomarker for HCC. However, our report has its limitation. Because of the lack of fund and patient cohorts, Hedgehog pathway and circ_0036412 were investigated mainly in cell lines instead of the HCC patients’ samples. In the future, we will conduct clinicopathological analyses to further explore their associations with stages of HCC patients.

## Supplementary Information

**Additional file 1: Fig. S1.** (A-C) The efficiencies of si-PRDM1-1/2/3, si-POLR2A-1/2/3 and si-CTCF-1/2/3 were detected by qRT-PCR in HuH-7 and Hep 3B cells (One-way ANOVA, Tukey). (D) The efficiency of pcDNA3.1-PRDM1 was detected by qRT-PCR in 293 T cells (Student’s t test). (E) The efficiency of si-circ_0036412-1/2/3 was detected by qRT-PCR in HuH-7 and Hep 3B cells (One-way ANOVA, Tukey). (F) The efficiency of si-GLI2-1/2/3 in HuH-7 and Hep 3B cells was detected by qRT-PCR (One-way ANOVA, Tukey). (G) The efficiency of pcDNA3.1-circ_0036412 in 293 T and HuH-7 cells was detected by qRT-PCR (Student’s t test). **P < 0.01.

**Additional File 2: Fig. S2.** (A-G) Human Protein Atlas (www.proteinatlas.org) predicted the subcellular location of U2AF2, EIF4A3, NOP58, FBL, IGF2BP2, ELAVL1 and PTBP1. (H) The efficiency of si-ELAVL1-1/2/3 was assessed by qRT-PCR in HuH-7 and Hep 3B cells (One-way ANOVA, Tukey). (I) The efficiency of pcDNA3.1-GLI2 in HuH-7 cells was assessed by qRT-PCR (Student’s t test). **P < 0.01.

## Data Availability

Not applicable.

## Abbreviations

HCC: Hepatocellular carcinoma
circRNA: Circular RNA
qRT-PCR: Quantitative real time-polymerase chain reaction
FISH: Fluorescence in situ hybridization
ChIP: Chromatin immunoprecipitation
RIP: RNA binding protein immunoprecipitation
RFA: Radiofrequency ablation
MADD: Multiple Acyl-CoA dehydrogenase deficiency
PTCH: Patched
SMO: Smoothened
FBS: Fetal bovine serum
siRNAs: Small interfering RNAs
cDNA: Complementary DNA
RNase R: Ribonuclease R
ActD: Actinomycin D
PVDF: Polyvinylidene fluoride
EdU: 5-Ethynyl-2′-deoxyuridine
TUNEL: Terminal deoxynucleotidyl transferase dUTP nick end labeling
CCK-8: Cell counting kit-8
DIG: Digoxigenin
SD: Standard deviation
ANOVA: Analysis of variance
GEO: Gene expression ominibus
RBPs: RNA-binding proteins
