# Supplementary figures and images for: Circ_0036412 affects the proliferation and cell cycle of hepatocellular carcinoma via hedgehog signaling pathway
Source: J Transl Med. 2022 Apr 5;20:154. doi: 10.1186/s12967-022-03305-x (PMC8981839; doi:10.1186/s12967-022-03305-x)

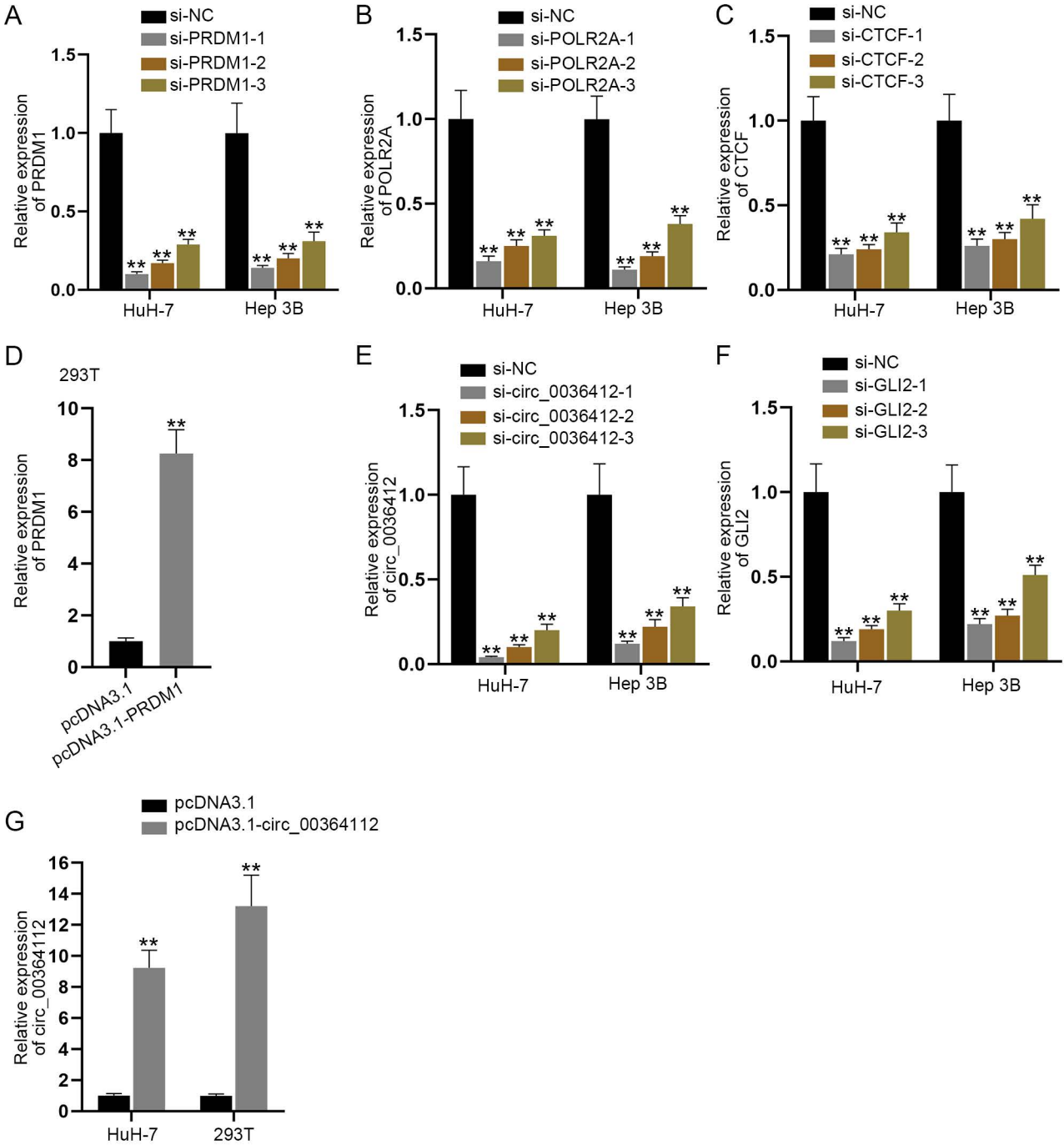

Supplement: Supplementary file 1 — Additional file 1: Fig. S1. (A-C) The efficiencies of si-PRDM1-1/2/3, si-POLR2A-1/2/3 and si-CTCF-1/2/3 were detected by qRT-PCR in HuH-7 and Hep 3B cells (One-way ANOVA, Tukey). (D) The efficiency of pcDNA3.1-PRDM1 was detected by qRT-PCR in 293 T cells (Student’s t test). (E) The efficiency of si-circ_0036412-1/2/3 was detected by qRT-PCR in HuH-7 and Hep 3B cells (One-way ANOVA, Tukey). (F) The efficiency of si-GLI2-1/2/3 in HuH-7 and Hep 3B cells was detected by qRT-PCR (One-way ANOVA, Tukey). (G) The efficiency of pcDNA3.1-circ_0036412 in 293 T and HuH-7 cells was detected by qRT-PCR (Student’s t test). **P < 0.01. [file 12967_2022_3305_MOESM1_ESM.pdf]

A

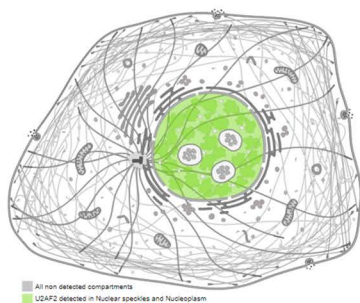

U2AF2

B

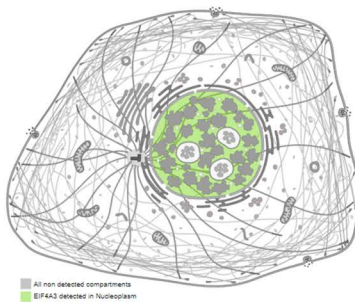

EIF4A3

C

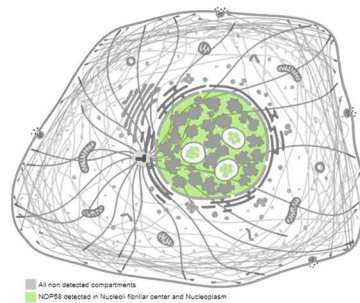

NOP58

D

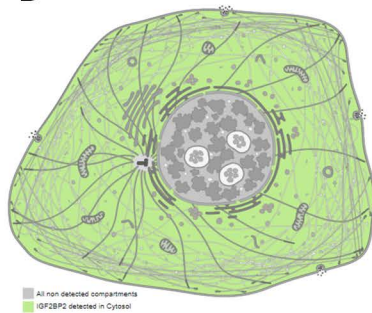

FBL

E

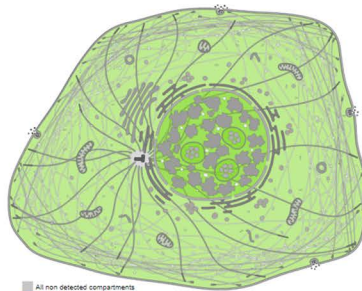

IGF2BP2

F

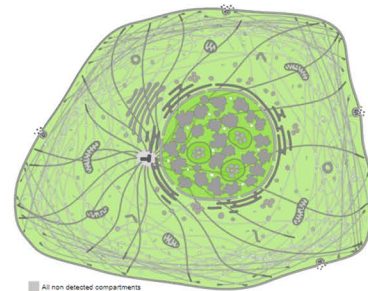

ELAVL1

G

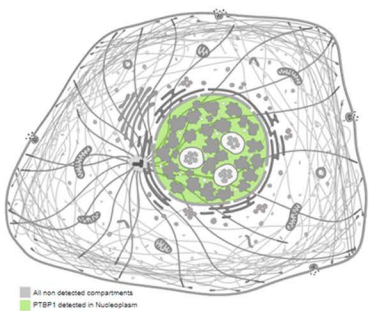

PTBP1

H

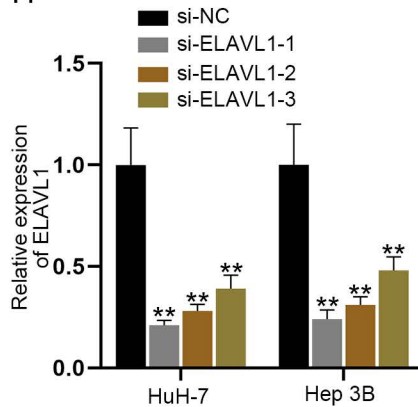

I

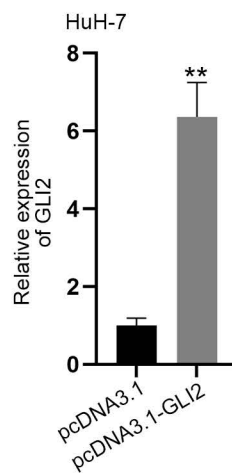

Supplement: Supplementary file 2 — Additional File 2: Fig. S2. (A-G) Human Protein Atlas (www.proteinatlas.org) predicted the subcellular location of U2AF2, EIF4A3, NOP58, FBL, IGF2BP2, ELAVL1 and PTBP1. (H) The efficiency of si-ELAVL1-1/2/3 was assessed by qRT-PCR in HuH-7 and Hep 3B cells (One-way ANOVA, Tukey). (I) The efficiency of pcDNA3.1-GLI2 in HuH-7 cells was assessed by qRT-PCR (Student’s t test). **P < 0.01. [file 12967_2022_3305_MOESM2_ESM.pdf]
